# Healthcare Disparities in Orthopaedic Surgery: A Comparison of Anterior Cruciate Ligament Reconstruction Incidence Proportions With US Census–Derived Demographics

**DOI:** 10.5435/JAAOSGlobal-D-22-00271

**Published:** 2023-07-06

**Authors:** Justin K. Solarczyk, Heather J. Roberts, Stephanie E. Wong, Derek T. Ward

**Affiliations:** From the University of California San Francisco School of Medicine, San Francisco, CA (Mr. Solarczyk); the Department of Orthopaedic Surgery, Mayo Clinic, Rochester, MN (Dr. Roberts); and the Department of Orthopaedic Surgery, University of California at San Francisco, Francisco, CA (Dr. Wong and Dr. Ward).

## Abstract

Disparities exist and affect outcomes after anterior cruciate ligament (ACL) injury. The purpose of this study was to investigate the association between race, ethnicity, and insurance type on the incidence of ACL reconstruction in the United States.

**Methods:**

The Healthcare Cost and Utilization Project database was used to determine demographics and insurance types for those undergoing elective ACL reconstruction from 2016 to 2017. The US Census Bureau was used to obtain demographic and insurance data for the general population.

**Results:**

Non-White patients undergoing ACL reconstruction with commercial insurance were more likely to be younger, male, less burdened with comorbidities including diabetes, and less likely to smoke. When we compared Medicaid patients who had undergone ACL reconstruction with all Medicaid recipients, there was an under-representation of Black patients and a similar percentage of White patients undergoing ACL reconstruction (*P* < 0.001).

**Discussion:**

This study suggests ongoing healthcare disparities with lower rates of ACL reconstruction for non-White patients and those with public insurance. Equal proportions of patients identifying as Black undergoing ACL reconstruction as compared with the underlying general population suggests a possible narrowing in disparities. More data are needed at numerous points of care between injury, surgery, and recovery to identify and address disparities.

In April 2021, the US Centers for Disease Control and Prevention issued a public statement declaring that racism is a serious threat to public health.^1^ A confluence of factors at the levels of individuals, institutions, and systems results in a racially and ethnically dependent differential access to resources, one of which is access to health insurance coverage and quality care.^2,3^ Healthcare inequalities continue to persist despite previous legislation, such as the Affordable Care Act, and subsequent policies designed to expand public health insurance.^3-7^

Race, ethnicity, sex, and socioeconomic status have been previously reported to affect access, quality, and outcomes of orthopaedic surgery.^8^ National, population-level data have identified these disparities in a broad range of orthopaedic topics including total hip and knee arthroplasty, shoulder arthroplasty, and care of the pediatric orthopaedic patient.^9-12^ Previously, a population-level, national investigation on anterior cruciate ligament (ACL) incidence and trends showed an increasing number of ACL reconstructions between 1996 and 2004, making this one of the most common orthopaedic surgeries conducted.^13^ Prior investigations of possible disparities in patients receiving surgical treatment of their ACL tears have been single-center, geographically restricted or restricted to a certain age group, insurance status, or gender demographic.^14-21^ There has yet to be a national, population-level multipayer analysis of racial, ethnic, and gender disparities in patients receiving surgical treatment of their ACL tears.

The purpose of this study was to identify whether there is a difference in access to specialized physicians and treatment by race, ethnicity, and insurance type in patients with ACL tears. Our hypothesis was that non-Hispanic White male status is associated with increased access to specialized physicians and treatment relative to Black and Hispanic patients, independent of insurance status.

## Methods

The Healthcare Cost and Utilization Project (HCUP) database maintained by the Agency for Healthcare Research and Quality was used to determine demographics and insurance types for those undergoing elective ACL reconstruction in Maryland, Florida, Iowa, and Wisconsin from 2016 to 2017. These states were chosen based on data availability and regional representativeness. The US Census Bureau for those states during the same period was used to obtain demographic and insurance data for the general population of the selected HCUP states.^22,23^

Patients undergoing ACL reconstruction were identified by Current Procedural Terminology code 29888 in HCUP data. Patients who were transferred from an outside facility or entered through the emergency department for their surgery were excluded to isolate elective cases. The proportions of patients in each race and ethnicity group and insurance type undergoing ACL reconstruction in the HCUP database were compared with the associated states' demographic data and insurance types from US Census data. Subgroup analysis by insurance type of the HCUP data was conducted. More granular demographic census data were limited for commercially insured and were unavailable for directly purchased commercial insurance, Tricare, and worker's compensation. In addition, because ACL reconstruction is more commonly conducted in younger patients, subgroup analysis was conducted for nonelderly patients (younger than 65 years). Because US Census data do not provide demographic information stratified by age for Medicare recipients, this group was excluded from the nonelderly subgroup analysis. Two-sample equality of proportion tests, chi square tests, and Student *t-*tests were used to analyze associations. A *P*-value of <0.05 was considered significant. Statistical analyses were conducted using Stata 16.0 (StataCorp). Institutional review board approval was not required because HCUP data are obtained from a deidentified database.

## Results

We identified 28,153 patients in Wisconsin, Iowa, Maryland, and Florida who underwent elective ACL reconstruction from 2016 to 2017. According to US Census data, the combined population of these states from 2016 to 2017 was 35,760,030. Compared with census data, the distributions of proportions of each race and ethnicity group in the ACL reconstruction cohort was markedly different, with a slightly higher proportion of Whites; a slightly lower proportion of Hispanics, Asians, and Native Americans; and a similar proportion of Blacks and African Americans (*P* < 0.001) (Table 1). The primary payer was more likely to be commercial insurance and less likely to be Medicare or Medicaid (*P* < 0.001) (Table 1).

**Table 1:** Demographics of ACL Healthcare Cost and Utilization Project Sample and Census

	ACL Reconstruction (n = 28,153)	Census (n = 35,760,030)	*P* Value
Age, 65 years and older: n (%)	147 (0.5%)	6,043,445 (16.9%)	<0.001
Female: n (%)	11,628 (41%)	18,208,685 (51%)	<0.001
Race: n (%)			
*White*	18,949 (80%)	27,704,299 (77%)	<0.001
*Black or African American*	3,249 (14%)	4,909,254 (14%)	
*Asian*	509 (2%)	1,374,517 (4%)	
*Native American*	120 (1%)	136,845 (<1%)	
Hispanic or Latino origin of any race: n (%)	3,877 (14%)	6,396,545 (18%)	<0.001
Primary payer: n (%)			
Commercial^a^	21,181 (84%)	19,733,278 (55%)	<0.001
*Medicare*	367 (1%)	5,598,942 (16%)	
*Medicaid*	3,364 (13%)	6,676,191 (19%)	
*Uninsured*^b^	415 (2%)	3,480,817 (10%)	

ACL = anterior cruciate ligament

a Commercial payer for the study cohort includes commercial or private insurance and for census includes employer and nongroup private insurance.

b Uninsured from the study cohort includes no charge and self-pay. Uninsured for census includes those without health insurance and those who have coverage under the Indian Health Service only.

Of patients with commercial insurance who underwent ACL reconstruction, 15,132 were White and 6,048 were non-White, with non-White patients more likely to be slightly younger (*P* < 0.001) and male (*P* < 0.001), and were less likely to be burdened with comorbidities (*P* < 0.001), to smoke (*P* < 0.001), and to have diabetes (*P* < 0.001) (Table 2). Similarly, non-White patients with Medicaid insurance who underwent ACL reconstruction were more likely to be younger (*P* < 0.001) and male (*P* < 0.001) and were less likely to be burdened with comorbidities (*P* < 0.027), to smoke (*P* < 0.001), and to have diabetes (*P* < 0.001) (Table 2).

**Table 2:** Characteristics of White and Non-White Patients Undergoing ACL Reconstruction by Commercial, Medicare, and Medicaid Insurance Status

	Commercial			Medicare			Medicaid		
	White (n = 15,132)	Non-White (n = 6,048)	*P* Value	White (n = 275)	Non-White (n = 92)	*P* Value	White (n = 1,562)	Non-White (n = 1,802)	*P* Value
Age, years: mean ± SD	29.4 ± 12.8	27.5 ± 11.2	<0.001	53.5 ± 15.3	43.5 ± 14.6	<0.001	25.7 ± 11.2	21.1 ± 8.6	<0.001
Age, younger than 18 years: n (%)	3,340 (22.1)	1,299 (21.5)	0.346	2 (0.7)	4 (4.4)	0.018	555 (35.5)	946 (52.5)	<0.001
Female: n (%)	6920 (45.7)	2,068 (34.2)	<0.001	132 (48.0)	48 (52.2)	0.488	704 (45.1)	678 (37.6)	<0.001
CCI: mean ± SD (CI)	0.41 ± 1.18 (0.39-0.43)	0.25 ± 0.99 (0.23-0.28)	<0.001	2.04 ± 2.16 (1.78-2.30)	2.07 ± 2.72 (1.50-2.63)	0.936	0.49 ± 1.38 (0.42-0.56)	0.39 ± 1.22 (0.33-0.44)	0.027
Required inpatient stay: n (%)	560 (3.7)	233 (3.9)	0.599	25 (9.1)	12 (13.0)	0.276	99 (6.3)	110 (6.1)	0.779
Smokers: n (%)	848 (5.6)	225 (3.7)	<0.001	56 (20.4)	25 (27.2)	0.173	256 (16.4)	161 (8.9)	<0.001
Diabetes: n (%)	566 (3.7)	142 (2.4)	<0.001	34 (12.4)	22 (23.9)	0.008	85 (5.4)	76 (4.2)	0.097

ACL = anterior cruciate ligament, CCI = Charlson Cororbidity Index, SD = standard deviation, C = 95% confidence interval.

Medicare patients who underwent ACL reconstruction were significantly more likely to be male (*P* < 0.001) compared with the general population with Medicare insurance (Table 3). No notable differences were observed in distribution of race or ethnicity among Medicare patients undergoing ACL reconstruction compared with the general population with Medicare insurance.

**Table 3:** Demographics of Medicare Recipients for ACL Healthcare Cost and Utilization Project Sample and Census

	ACL Reconstruction (n = 367)	Census (n = 6,705,900)	*P* Value
Age, 65 years and older: n (%)	114 (31.1)	5,921,310 (88.3)	<0.001
Female: n (%)	180 (49.1)	3,735,150 (55.7)	<0.001
Race: n (%)			
*White*	275 (77.9)	5,158,200 (76.9)	0.077
*Black or African American*	45 (12.8)	682,300 (10.2)	
*Hispanic*	31 (8.8)	722,800 (10.8)	
*Asian*	2 (0.6)	125,400 (1.9)	

ACL = anterior cruciate ligament

A subgroup analysis of nonelderly patients younger than 65 years was conducted for patients with either Medicaid or commercial insurance. Black patients represent 23% of the total nonelderly Medicaid recipients but comprise 29% of Medicaid patients undergoing ACL reconstruction (*P* < 0.001) (Table 4). Compared with the nonelderly general population with commercial insurance, those undergoing ACL reconstruction with commercial insurance were more likely to be White or Hispanic and less likely to be Black or Asian (*P* < 0.001) (Table 4). In both Medicaid and commercial insurance groups, the age distribution of patients who underwent ACL reconstruction was skewed toward younger age groups compared with the general population, with 6 to 17 years and 18 to 24 years particularly over-represented.

**Table 4:** Demographics of Nonelderly Medicaid and Commercial Insurance Recipients for ACL Healthcare Cost and Utilization Project Sample and Census

	Medicaid			Commercial		
	ACL Reconstruction (n = 3,177)	Census (n = 5,643,321)	*P* Value	ACL Reconstruction (n = 20,103)	Census^a^ (n = 16,559,775)	*P* Value
Age, year: n (%)						
*Less than 6*	1 (<1)	1,075,146 (19.1)	<0.001	1 (<1)	1,119,978 (6.8)	<0.001
*6-17*	1,395 (43.9)	1,929,768 (34.2)		4,383 (21.8)	2,773,006 (16.7)	
*18-24*	700 (22.0)	450,489 (8.0)		5,165 (25.7)	1,811,419 (10.9)	
*25-34*	552 (17.4)	612,978 (10.9)		4,263 (21.2)	2,450,219 (14.8)	
*35-44*	371 (11.7)	523,078 (9.3)		3,391 (16.9)	2,631,213 (15.9)	
*45-54*	129 (4.1)	490,444 (8.7)		2,210 (11.0)	2,985,593 (18.0)	
*55-64*	30 (0.9)	561,420 (9.9)		691 (3.4)	2,788,349 (16.8)	
Female: n (%)	1,308 (41.2)	3,006,722 (53.3)	<0.001	8,587 (42.7)	8,361,227 (50.5)	<0.001
Race: n (%)			<0.001			<0.001
*White*	1562 (49.2)	2,799,087 (49.6)		15,116 (75.2)	11,807,120 (71.3)	
*Hispanic*	625 (19.7)	1,049,658 (18.6)		2,622 (13.0)	1,639,418 (9.9)	
*Black or African American*	920 (29.0)	1,314,894 (23.3)		1,864 (9.3)	2,053,412 (12.4)	
*Asian*	46 (1.5)	174,943 (3.1)		416 (2.1)	678,951 (4.1)	

ACL = anterior cruciate ligament

a Either primary beneficiaries or as a dependent of employer-sponsored health coverage

## Discussion

This is the first nationally representative database study investigating the influence of race, ethnicity, and insurance type on access to specialized physicians and treatment through elective ACL reconstruction. We identified several differences between the general population and patients undergoing ACL reconstruction. Overall, patients who underwent ACL reconstruction were more likely to be younger, male, and non-Hispanic White and have commercial insurance compared with the underlying general population. Indeed, these data suggest that non-Hispanic White patients did not have a greater proportion of ACL reconstruction compared with other races and ethnicities, independent of insurance status. While the overall proportion of Black or African American (AA) patients undergoing ACL reconstruction was similar to the proportion of Black or AA patients in the general population, we found notable interactions between race, ethnicity, and insurance status that suggest that disparities persist in access to surgical care.

Previous studies similarly show that the age distribution of patients undergoing ACL reconstruction tend to be younger than the general population.^16,17,24,25^ While US Census data only stratify by age younger or older than 65 years for patients with Medicare insurance, limiting our ability to further investigate age trends in ACL reconstruction, we similarly show that patients with Medicare insurance undergoing ACL reconstruction are markedly more likely to be younger than 65 years than the general population. Among those with Medicaid and commercial insurance, the age distribution of patients undergoing ACL reconstruction is skewed toward younger patients compared with the general population, which is consistent with previous data reported by Herzog et al and Sander et al^17,20^ who reported the highest absolute rates of ACL reconstruction in the age group of 18 to 30 years. There are many possible factors that help explain why younger people undergo ACL reconstruction more often than older patients; however, it is possible that younger people are more likely to be in school and may have improved screening when compared with older patients.^16,17,20,24,25^

Studies have shown that patients with an ACL injury who have public health insurance face obstacles in obtaining specialty care compared with patients with commercial insurance and that public health insurance is an independent risk factor of lower rates of access to ACL reconstruction.^14-16,19,26,27^ More specifically, as reported by Baraga et al and later similarly reported by Khanna et al, ACL reconstruction patients with commercial insurance were 2.15 times more likely to be correctly diagnosed within a given period compared with those with public insurance, after adjusting for age, sex, and employment status.^14,18^ A study by Williams et al^28^ concluded that adolescents with ACL injuries who had public insurance presented with higher rates of chondral injuries and irreparable meniscus tears, likely indicating barriers to timely access to specialty care. Limitations in access to advanced imaging for accurate diagnosis of ACL injuries may also play a role because Hispanic patients have decreased access to hospitals with advanced imaging capabilities compared with White non-Hispanic patients.^29^ Our data, which show that 13% of patients undergoing ACL reconstruction have Medicaid insurance compared with 19% of the general population with Medicaid insurance, may suggest persistent disparities in access to care among patients with public insurance and replicate earlier published data by Dodwell et al^16^ that public insurance is associated with lower rates of ACL reconstruction.

Previous studies suggest that Black and Hispanic pediatric patients are more likely to use public health insurance for ACL reconstruction, and White pediatric patients are more likely to use commercial insurance for ACL reconstruction.^15^ We similarly show that of Medicaid recipients undergoing ACL reconstruction, the proportion of pediatric patients identifying as a non-White race or ethnicity was sixfold greater than the proportion of patients identifying as White.

Importantly, our database study identifies patients who underwent ACL surgery but is unable to identify patients with ACL injury or diagnosis, and we are, therefore, unable to determine whether differences in surgical management are because of differences in rates of ACL tear by race, ethnicity, or insurance status. There is scarce literature describing racial or ethnic differences in rates of ACL injury. In a population of professional women basketball players, Trojian et al observed that White European American women had an ACL tear rate six times that of all other races or ethnicities combined.^30^ Although the reason for this difference in rate of ACL tear was not determined in this study, which had a sample size of 371, the authors speculated that it could be because of anatomy, specifically differences in intercondylar shelf angle and anterior curvature of the femur. Our study showed that the proportion of Black or African American patients undergoing ACL reconstruction is representative of the general population. It is possible that this is an indication of progress in the narrowing of healthcare disparities; however, many factors are at play. Between potential differences in ACL tear incidence, accurate diagnosis, subspecialty referral and decision for surgery, and subsequently ACL reconstruction surgery, it is challenging to elucidate exactly how and whether racial disparities are being combatted successfully.

We can compare our findings of racial and ethnic differences in ACL reconstruction with other orthopaedic and nonorthopaedic surgical procedures. Previous literature has provided evidence of persistent and worsening racial disparities in orthopaedic and nonorthopaedic surgical use, even after controlling for insurance status, geography, and hospital teaching status from 2012 to 2017.^31^ The surgery types ranged from coronary artery bypass graft (CABG), to colorectal resection, to total hip arthroplasties. We replicate these findings, and most notably, we find that Black patients comprise 12% of the total population with commercial insurance, although only 9% of those are with commercial insurance who undergo ACL reconstruction. Yet, these findings are to be contrasted against our data showing that although Black patients represent 23% of total nonelderly Medicaid recipients, they comprise 29% of Medicaid patients undergoing ACL reconstruction.

We must work to narrow healthcare disparities at all levels of care.^8^ At the highest levels, introducing legislation to raise rates of reimbursement for Medicaid would incentivize providers to see Medicaid patients and provide timely care. All the way down to the physician and patient interaction level, physicians must be sure to take their biases into account during clinical decision making to ensure equitable care.

This study has several limitations. We used a large nationally representative database, and our data are subject to limitations inherent to database studies, such as errors in coding of diagnoses and procedures. Four states were selected, which may limit generalizability, and our states' percentages of minorities adequately approximate national census estimates 2016 to 2017.^23^ The commercial insurance group from the US Census data did not include worker's compensation or personally purchased commercial insurance. However, because only 7% of the population purchases commercial insurance, this is unlikely to markedly affect our analysis.^22^ As discussed earlier, we were unable to determine rates of ACL injury by race, ethnicity, and insurance status. We were also unable to assess any information regarding participation in sports within younger populations, which could possibly inform questions regarding access to MRI, accurate diagnosis from a physician, and willingness to undergo treatment. Finally, we were unable to assess timeliness of surgical management, incidence of additional intra-articular pathology, or postoperative outcomes.

In conclusion, our study may suggest ongoing healthcare disparities with lower rates of ACL reconstruction for non-White patients and those with public insurance. To clarify the possible presence of these disparities, additional research is needed to characterize factors such as differences in ACL tear incidence, accurate diagnosis, subspecialty referral and decision for surgery, and, subsequently, ACL reconstruction surgery. Some of our data suggest a possible narrowing in healthcare disparities, with equal proportions of patients identifying as Black undergoing ACL reconstruction as compared with the underlying general population. More data are needed at the numerous points of care between injury, surgery, and recovery to identify and address disparities.
